# Paranormal belief and well-being: The moderating roles of transliminality and psychopathology-related facets

**DOI:** 10.3389/fpsyg.2022.915860

**Published:** 2022-08-15

**Authors:** Neil Dagnall, Andrew Denovan, Kenneth Graham Drinkwater, Álex Escolà-Gascón

**Affiliations:** ^1^Department of Psychology, Manchester Metropolitan University, Manchester, United Kingdom; ^2^Department of Psychology, University of Huddersfield, Huddersfield, West Yorkshire, United Kingdom; ^3^Department of Applied Mathematics and Statistics, Universitat Ramon Llull, Barcelona, Catalonia, Spain

**Keywords:** belief in the paranormal, schizotypy, well-being, moderation, transliminality, manic-depressive experience

## Abstract

Evaluation of prior research suggests that belief in the paranormal is more likely to be associated with negative psychological functioning, when presented alongside cognitive-perceptual factors that askew thinking and insight. The current study examined this notion using a sample of 3,084 participants (1,382 males, 1,693 females, nine non-binary). Respondents completed self-report measures assessing Paranormal Belief, Transliminality, psychopathology-related characteristics (Schizotypy and Manic-Depressive Experience), and well-being (Perceived Stress and Somatic Complaints). Responses were analysed *via* correlations and moderation. Paranormal Belief correlated positively with Transliminality, psychopathology-related measures, Perceived Stress, and Somatic Complaints. Moderation analyses revealed that Transliminality and psychopathology-related variables (i.e., the Unusual Experiences and Cognitive Disorganisation subscales of schizotypy, and Manic-Depressive Experience) interacted with Paranormal Belief in complex ways and were allied to higher scores on negative well-being outcomes. This indicated that within paranormal believers, Transliminality and specific psychopathology-related variables in combination predicted susceptibility to negative well-being outcomes.

## Introduction

Academic surveys (Dagnall et al., 2016b) and opinion polls (Ipsos MORI, 1998, 2003; Newport and Strausberg, 2001; Moore, 2005) report that belief in the paranormal prevails within modern Western societies. Despite investigators using varying definitions of paranormal belief and measurement instruments differing in construct content, endorsement is consistently cited at around 50% (Irwin, 2009; Marks, 2021). Acknowledging these conceptual issues, the present study adopted the classification proposed by Irwin (2009), which states that “a paranormal belief is defined on a working basis as a proposition that has not been empirically attested to the satisfaction of the scientific establishment but is generated within the nonscientific community and extensively endorsed by people who might normally be expected by their society to be capable of rational thought and reality testing” (Irwin, 2009, p. 16–17).

The relatively high incidence of supernatural credence observed across studies, surveys, and polls indicates that paranormal belief is an important construct, which merits psychological investigation. Noting this, researchers have evaluated the function and effects of paranormal belief on outcomes such as well-being (Irwin, 2000). In this context, investigators have frequently observed a positive relationship between belief in the paranormal and higher levels of psychopathological symptoms (i.e., depressive, Thalbourne and French, 1995; manic, Thalbourne and French, 1995; and psychotic, Dag, 1999; Peltzer, 2002; Thalbourne and Storm, 2019; Liu et al., 2021).

Commensurate with these findings, studies report that superstition (a specific facet of paranormal belief), which embodies the conviction supernatural forces such as fate and luck effect real-world events, is associated with poor psychological adjustment (e.g., high neuroticism, Vyse, 2013; low self-efficacy, Tobacyk and Shrader, 1991; and irrational beliefs, Roig et al., 1998; Dagnall et al., 2007, 2009; Wiseman and Watt, 2004; Hoffmann et al., 2022). Collectively, these findings imply that belief in the paranormal is directly related to lower levels of well-being and poorer psychological adjustment (i.e., higher negative emotional states and a depressive attributional style; Dudley and Whisnand, 2000).

Irwin (2009) explicates the non-adaptive effects of paranormal belief in terms of psychodynamic functions, whereby magical thinking serves as a strategy to address lack of perceived control and resolve uncertainty. From this perspective, belief in the paranormal provides a framework for imposing meaning and structure on the world. Thus, supernatural explanations reduce anxiety by providing believers with a sense of illusory control (Irwin, 1993, 2003, 2009). However, since psychological benefits are domain specific (Roe and Bell, 2016), paranormal belief generally is reflective of poor psychological functioning. Corresponding with this notion, McGarry and Newberry (1981) reported that believers typically view the world as unpredictable, problem-laden, difficult, and unjust.

Recent academic work on stress management has suggested that the use of scientifically unsubstantiated beliefs as a mechanism for resolving life pressures is an ineffective strategy (Marchlewska et al., 2021). This concurs with Irwin (1991), who found a negative correlation between belief in the paranormal and psychological coping. This is explained by the fact that endorsement of scientifically unsubstantiated theories is a form of avoidance response, whereby individuals use beliefs to circumvent dealing with challenging circumstances and feelings. This strategy is maladaptive since it encourages withdrawal from goal-related behaviours (Mackay et al., 2011). Hence, avoidance is related positively to adverse consequences (lower well-being, depression, powerlessness, and addiction; Sagone and De Caroli, 2014).

Avoidance contrasts with approach strategies, which actively address stressful situations and allied emotions. Approach is adaptive because it facilitates goal attainment and the positive affect through adoption of a problem-focused orientation, acceptance of situational demands, active solution seeking, and engagement with social support (Fortune et al., 2002). Accordingly, approach strategies are negatively associated with undesirable outcomes (depression and functional disability; Greenglass et al., 2006).

Despite these findings, studies have typically failed to observe consistent effects. Illustratively, some investigators have observed no significant relationships between belief in the paranormal and well-being (Willging and Lester, 1997). Moreover, other researchers propose that supernatural beliefs are conducive to positive mental health. The notion that paranormal credence is beneficial concurs with scholarly work on religion. This advocates that despite often confounding critical scrutiny, beliefs provide high order adaptive functions (Kanazawa, 2015). In the case of the paranormal, Schumaker (1990) contends that beliefs serve as reality shelters (Schumaker, 1990). Consistent with this supposition, Schumaker (1987) reported that belief in the paranormal correlated negatively with severity of psychopathology. Irwin (1991), however, reported contradictory findings.

Further support for the beneficial effects of paranormal belief was provided by Betsch et al. (2021), who observed that paranormal belief played a positive role in the development of self-concept. Analysis of interview narratives revealed that beliefs provided subjective, generic theories of the world that helped individuals to identify the purpose of their lives. These conclusions aligned with Drinkwater et al. (2017), who performed thematic analysis on personal accounts of subjective paranormal experiences and found they were also associated with the desire to comprehend the external environment; specifically resolve uncertainty, and construct meaning.

The personal benefits of paranormal experiences were further highlighted by Drinkwater et al. (2013). Using interpretative phenomenological analysis, they noted that descriptions of paranormal experiences were often accompanied by positive affect, which enhanced sense of well-being and spirituality. Explicitly, the belief that paranormal phenomena represented evidence of life after death enabled interviewees to resolve emotional conflicts arising from loss. The potential psychological benefits of paranormal advocacy are not merely limited to belief and experience. In a recent study using reflexive thematic analysis, Drinkwater et al. (2022a,b) found that justification of professed paranormal abilities created a sense of personal coherence and significance by providing a framework for structuring significant life events.

Collectively, these studies suggest that paranormal endorsement in some circumstances can prove personally adaptive. For example, when people have near death experiences, if the event is successfully integrated into lived experience, it can have overwhelmingly positive psychological outcomes (e.g., enhanced appreciation of life, positive attitude towards self, and enhanced sense of self-identity; Tassell-Matamua and Lindsay, 2016). Whereas in other instances, paranormal endorsement reflects negative psychological states such as anxiety and ontological confusions (i.e., confounding core aspects of reality; conflation of mental with physical phenomena; Lindeman et al., 2015). Moreover, when beliefs are challenged and refuted by others they can be detrimental to self (Betsch et al., 2021).

Given the relatively high prevalence of paranormal belief within the general population it is reasonable to assume that supernatural credence *per se* is only non-adaptive in particular instances (Dagnall et al., 2016b; Drinkwater et al., 2018; Williams et al., 2021). For example, when beliefs become rigid and/or exert a powerful influence on the individual that their everyday functioning is impaired. In such circumstances beliefs can become pathological and harmful to well-being. At a social level, paranormal beliefs have detrimental effects on individual and collective welfare when they combine with destructive dogmas (e.g., witch hunts; Tobacyk, 1983).

Hence, in the absence of heightened scores on related cognitive-perceptual factors, paranormal belief appears relatively benign. This explains why even within studies that report relationships between belief in the paranormal and negative well-being, associations are typically weak/moderate. Furthermore, when investigations have examined only direct relationships, it is impossible to determine whether supernatural endorsement is the cause or consequence of poor psychological adjustment. Acknowledging this, belief in the paranormal is more likely related to negative psychological functioning when it is concomitant with cognitive-perceptual personality factors that askew thinking and insight (e.g., schizotypy; Irwin et al., 2012a,b). Specifically, when interactions produce non-adaptive beliefs that adversely influence perception of the external world (Drinkwater et al., 2021).

One construct that is likely to moderate the relationship between psychopathological indicators (i.e., schizotypy and manic-depressive experience) is transliminality. Transliminality represents the “hypothesized tendency for psychological material to cross (trans) thresholds (limines) into or out of consciousness” (Thalbourne and Houran, 2000, p. 853). In this context, level of transliminality is important because it influences the “experienced” flow of sensory information (Thalbourne, 2009a). Consistent with this perspective, high transliminality is conceptualized as a “permeable” mental threshold (Thalbourne, 1998) that allows greater levels of material from unconscious (endo-psychic) and external sources to enter awareness (Thalbourne and Maltby, 2008). In the case of believers, this provides a steady stream of anomalous perceptions that can be labelled as supernatural (Rock et al., 2021). Accordingly, theorists view transliminality as predictive of the general trait tendency to hold paranormal beliefs (Dagnall et al., 2010a; Rock et al., 2021).

Concomitantly, hypersensitivity to threatening stimuli is associated with vulnerability to psychosis, depression, and mania (Calvo et al., 2021). Correspondingly, transliminality is likely to interact with psychopathology-related facets by virtue of this relationship. This may occur because involuntary susceptibility to ideational phenomena increases the likelihood of experiencing negative outcomes (e.g., heightened stress and unpleasant affect; Kreiselmaier, 2016). The notion that interactions between paranormal belief and cognitive-perceptual personality factors influence well-being, extends also to experience (Escolà-Gascón, 2020a,b). For instance, Escolà-Gascón et al. (2021) found that when clinical features and psychopathological risks were identified in believers, then experiences were also psychopathological in nature. Explicitly, they were generated by magical or irrational belief systems (Irwin et al., 2013).

This implies that interpreting an everyday experience as “paranormal” is an attribution that feeds back into the belief system (Escolà-Gascón, 2022). Acknowledging these factors, this study appraised the supposition that cognitive-perceptual factors that characterise idiosyncratic mentation (transliminality, schizotypy, manic-depressive experience) moderate (strengthen) relationships between paranormal belief and negative facets of well-being (i.e., higher stress and somatic complaints).

## Materials and methods

### Participants

In total, 3,084 respondents participated (Mage = 48.96, SD = 15.01, range 18–83). The sample comprised 1,382 males (Mage = 54.84, SD = 13.51, range 18–83), 1,693 females (Mage = 44.20, SD = 14.45, range 18–83), and 9 non-binary respondents (Mage = 40.00, SD = 18.13, range 19–78). The researchers used a United Kingdom-based sample with a minimum age of 18 years provided by Bilendi (an established provider of representative online samples; Salak et al., 2021). Such data is high quality and comparable with that collected *via* orthodox methods (Kees et al., 2017).

### Measures

#### Paranormal belief

The Revised Paranormal Belief Scale (RPBS; Tobacyk, 2004) assessed paranormal belief. The measure contains 26-items (e.g., “Reincarnation does occur”) accompanied by a seven-point Likert scale (0 = strongly disagree to 6 = strongly agree; see Irwin, 2009). High scores reflect greater levels of belief. The measure possesses satisfactory validity and reliability (Drinkwater et al., 2017).

#### Transliminality

The Revised Transliminality Scale (RTS; Thalbourne et al., 1997; Thalbourne, 1998; Lange et al., 2000) measured the hypothesized tendency for psychological material to cross thresholds into or out of consciousness. Items (e.g., “Thoughts sometimes come too quickly to write down”) are presented as statements and answered in a “yes/no” format. Though 29-items are administered, 12-items (which remain as fillers) are omitted from scoring due to gender and age biases identified by Rasch analysis (see Houran et al., 2003). The Rasch version is psychometrically superior to the original (i.e., it possesses good internal and test–retest reliability; Houran et al., 2003). Higher scores reflect greater transliminality.

#### Psychopathology-related measures

##### Schizotypy

The Oxford-Liverpool Inventory of Feelings and Experiences (O-LIFEshort; Mason et al., 2005) is an abridged 43-item form of the O-LIFE (Mason et al., 1995) that captures schizotypal traits among non-clinical populations. Four subscales exist: Unusual Experiences, Cognitive Disorganisation, Introvertive Anhedonia, and Impulsive Non-Conformity. Unusual Experiences (12-items) measures positive schizotypy (magical thinking, perceptual irregularities, and hallucinations). Cognitive Disorganisation (11-items) assesses disorganized features of psychosis (e.g., poor concentration/attention). Introvertive Anhedonia (10-items) captures aspects of negative schizotypy involving avoidance of intimacy and withdrawal. Impulsive Non-Conformity (10-items) represents low self-control, antisocial and impulsive conduct. Items are administered as questions (e.g., “Is it hard for you to make decisions?”) alongside a “yes/no” format. The total scale demonstrated good internal reliability; subscale alpha coefficients range from 0.62 to 0.80 (Mason et al., 2005).

##### Manic-depressiveness

The Manic-Depressiveness Scale (Thalbourne et al., 1994) contains two 9-item “true/false” subscales: manic experience (e.g., “I have gone for more than a day with much less sleep than I normally needed and yet still not been tired”), and depressive experience (e.g., “I have in the past made active attempts to die”). The scale has established validity and reliability (see Lester, 2000).

#### Well-being

##### Perceived stress

The Perceived Stress Scale (PSS-10; Cohen and Williamson, 1988) assesses how uncontrollable and unpredictable individuals find their lives (focusing on the past month). The PSS-10 includes 10-items (e.g., “How often have you been angered because of things that were outside of your control?”), with a 0 (never) to 4 (very often) Likert scale. Acceptable reliability and validity exist for the PSS-10 (Denovan et al., 2019).

##### Somatic complaints

The Somatic Symptom Scale-8 (SSS-8) measures vulnerability to somatic complaints. Items capture the degree to which somatic ailments (e.g., “Feeling tired or having low energy”) have impacted respondents during a seven-day period. Respondent’s answer using a five-point Likert ranging from 0 (not at all) to 4 (very much). The SSS-8 possesses good internal reliability (Gierk et al., 2014).

### Procedure

Potential respondents clicked on a web link to retrieve the study information sheet (specifying the study’s objectives and participant rights). After reading this, only participants providing informed consent continued to the survey, which included a demographics section (i.e., preferred gender and age), and the measurement instruments. Scale order rotated across respondents to prevent order effects. After taking part, respondents were debriefed.

As the study was cross-sectional, procedural remedies were introduced to reduce potential common method variance (Krishnaveni and Deepa, 2013). Specifically, section instructions created psychological distance between scales by emphasizing construct differences. In addition, to address evaluation apprehension and social desirability respondents were instructed to read all statements carefully, work at their own speed, and answer all items. They were also told that there were no right or wrong answers.

### Ethics statement

The Manchester Metropolitan University Faculty of Health, Psychology and Social Care Ethics Committee granted ethical approval (December 2020; Project ID, 25390).

## Results

### Preliminary analysis

Data screening revealed no issues with normality, linearity, or multicollinearity (i.e., all correlations were below 0.9, and skewness and kurtosis between −2 and +2). No multivariate outliers existed; data values were >0.001 relative to Mahalonobis Distance and chi-square distribution (Tabachnick et al., 2007). All measures exhibited internal reliability coefficients >0.7, aside from Introvertive Anhedonia and Impulsive Non-Conformity (Table 1). These results aligned with prior work (see Mason et al., 2005).

**Table 1:** Descriptive statistics and correlations.

Variable	Mean	SD	Skew	Kurtosis	*α*	1	2	3	4	5	6	7	8	9	10
1. Paranormal Belief	58.90	33.44	0.02	−0.83	0.95		0.48^**^	0.55^**^	0.30^**^	0.02	0.27^**^	0.27^**^	0.29^**^	0.25^**^	0.33^**^
2. Transliminality	20.41	5.26	0.69	0.45	0.92			0.72^**^	0.51^**^	0.02	0.46^**^	0.65^**^	0.60^**^	0.32^**^	0.43^**^
3. Unusual Experiences	3.66	3.25	0.65	−0.53	0.85				0.58^**^	0.04^*^	0.48^**^	0.53^**^	0.52^**^	0.34^**^	0.41^**^
4. Cognitive Disorganisation	4.24	3.44	0.43	−0.99	0.86					0.21^**^	0.54^**^	0.54^**^	0.60^**^	0.54^**^	0.45^**^
5. Introvertive Anhedonia	3.39	2.09	0.25	−0.64	0.60						0.18^**^	0.08^**^	0.21^**^	0.23^**^	0.20^**^
6. Impulsive Non-Conformity	2.59	2.10	0.75	−0.06	0.65							0.47^**^	0.58^**^	0.47^**^	0.41^**^
7. Manic Experience	12.32	2.35	0.34	−0.70	0.80								0.70^**^	0.39^**^	0.41^**^
8. Depressive Experience	11.98	2.52	0.59	−0.64	0.71									0.56^**^	0.53^**^
9. Perceived Stress	17.27	7.75	0.13	−0.06	0.87										0.54^**^
10. Somatic Complaints	17.06	7.18	0.72	−0.19	0.86										

^*^p < 0.05; ^**^p < 0.001 (also less than the Benjamini and Hochberg adjusted critical *p* values).

Zero-order correlations (Table 1) revealed small to large positive associations between Paranormal Belief, Transliminality, psychopathology-related variables (Schizotypy factors and Manic and Depressive Experience), and well-being variables (Perceived Stress and Somatic Complaints).

For purposes of comparison, since analysis considered multiple correlations, adjustment to the significance level occurred. This used the sequential method suggested by Benjamini and Hochberg (1995) and demonstrated by Williams et al. (1999). Following this, a ranking of *p* values (from smallest to largest) took place, resulting in adjusted critical *p* values for statistical inference, according to the formula of I/K × 0.05 (i.e., observed *p* value rank/number of comparisons × level of significance). Comparisons employed the 0.05 significance level. This method regulates the false positive rate, ensuring that no more than 5% of results identified as significant are in the wrong direction.

### Moderation analysis

Hayes PROCESS macro for moderation analysis (Hayes, 2013) assessed whether Transliminality and psychopathology-related variables affected the strength and direction of the relationship between Paranormal Belief and well-being (Perceived Stress and Somatic Complaints). This macro runs a series of OLS regressions with the centred product term representing the interaction of designated predictor and moderator variables with specific criterion variables.

Analyses employed bootstrapping (5,000 resamples) to generate 95% bias-corrected confidence intervals. Initially, moderation of Transliminality occurred in relation to Paranormal Belief and well-being. Subsequently, assessment of the additive effect of psychopathology-related variables occurred. This involved testing the moderating role of several variables (Schizotypy factors: Unusual Experiences, Cognitive Disorganisation, Introvertive Anhedonia, Impulsive Non-Conformity; and Manic and Depressive Experience) in relation to Paranormal Belief, Transliminality, Perceived Stress and Somatic Complaints.

Paranormal Belief was a significant predictor of Perceived Stress, *b* = 0.03, 95% CI [0.02, 0.04], *t* = 6.29, *p* < 0.001 (Table 2). Transliminality was also a significant prognosticator, *b* = 0.41, 95% CI [0.35, 0.46], *t* = 13.71, *p* < 0.001. A significant Paranormal Belief × Transliminality interaction existed, *b* = −0.01, 95% CI [−0.02, −0.01], *t* = −3.65, *p* < 0.001. Scrutiny of the interaction (*via* simple slopes) inferred that the association between Paranormal Belief and Perceived Stress was significant at low (*t* = 7.33, *p* < 0.001), mean (*t* = 6.29, *p* < 0.001), and high (*t* = 2.38, *p* = 0.017) levels of Transliminality. This indicated that the higher the Transliminality, the greater the Paranormal Belief and Perceived Stress. The model accounted for 11.6% of variance in Perceived Stress. Paranormal Belief additionally predicted Somatic Complaints, *b* = 0.03, 95% CI [0.02, 0.04], *t* = 8.31, *p* < 0.001, as did Transliminality, *b* = 0.48, 95% CI [0.43, 0.53], *t* = 18.63, *p* < 0.001. A significant interaction did not occur, *b* = 0.01, 95% CI [−0.01, 0.01], *t* = 0.56, *p* = 0.575, indicating that Paranormal Belief and Transliminality independently predicted increased Somatic Complaints. In total, 20.6% of Somatic Complaints variance was accounted for.

**Table 2:** Transliminality and psychopathology-related variables as potential predictors/moderators of Perceived Stress and Somatic Complaints.

Model	Perceived Stress			Somatic Complaints		
	*b*	*t*	*p* (95% CI)	*b*	*t*	*p* (95% CI)
TR ± UE						
PB	0.007	1.26	0.204 (−0.004, 0.017)	0.016	3.43	<0.001 (0.006, 0.025)
TR	0.168	4.31	<0.001 (0.091, 0.244)	0.328	9.50	<0.001 (0.260, 0.395)
UE	0.620	9.38	<0.001 (0.490, 0.750)	0.382	6.54	<0.001 (0.267, 0.496)
PB × TR	0.001	0.35	0.722 (−0.002, 0.003)	0.001	0.597	0.550 (−0.001, 0.003)
PB × UE	−0.017	−3.30	<0.001 (−0.010, −0.002)	−0.001	−0.40	0.682 (−0.004, 0.003)
TR × UE	−0.030	−3.21	0.001 (−0.048, −0.011)	−0.026	−3.20	0.001 (−0.043, −0.010)
PB × TR × UE	0.010	2.88	0.004 (0.002, 0.010)	0.010	3.52	<0.001 (0.004, 0.010)
TR ± CD						
PB	0.021	4.89	<0.001 (0.013, 0.029)	0.026	6.42	<0.001 (0.018, 0.034)
TR	0.035	1.16	0.242 (−0.023, 0.092)	0.284	10.11	<0.001 (0.229, 0.339)
CD	1.17	28.47	<0.001 (1.09, 1.25)	0.605	15.48	<0.001 (0.529, 0.682)
PB × TR	−0.001	−0.38	0.702 (−0.002, 0.001)	0.000	0.24	0.805 (−0.001, 0.002)
PB × CD	−0.004	−3.08	0.002 (−0.007, −0.001)	0.002	1.81	0.069 (−0.001, 0.005)
TR × CD	−0.001	−1.28	0.198 (−0.025, 0.005)	−0.020	−2.84	0.004 (−0.035, −0.006)
PB × TR × CD	0.000	−0.06	0.945 (−0.001, 0.001)	0.010	2.29	0.021 (0.001, 0.010)
TR ± IA						
PB	0.028	6.31	<0.001 (0.019, 0.036)	0.032	8.38	<0.001 (0.024, 0.039)
TR	0.407	13.98	<0.001 (0.349, 0.464)	0.482	18.82	<0.001 (0.431, 0.532)
IA	0.859	12.63	<0.001 (0.726, 0.992)	0.714	11.94	<0.001 (0.597, 0.832)
PB × TR	−0.003	−4.27	<0.001 (−0.004, −0.002)	0.001	0.126	0.899 (−0.001, 0.001)
PB × IA	0.003	1.26	0.204 (−0.001, 0.007)	0.005	2.88	0.004 (0.002, 0.009)
TR × IA	0.001	0.66	0.505 (−0.019, 0.038)	0.023	1.86	0.061 (−0.001, 0.048)
PB × TR × IA	−0.000	−0.53	0.594 (−0.001, 0.001)	−0.001	−1.47	0.141 (−0.001, 0.001)
TR ± IN						
PB	0.021	6.31	<0.001 (0.013, 0.030)	0.028	7.08	<0.001 (0.020, 0.036)
TR	0.157	5.21	<0.001 (0.098, 0.216)	0.341	12.28	<0.001 (0.286, 0.395)
IN	1.600	23.16	<0.001 (1.465, 1.735)	0.899	14.16	<0.001 (0.774, 1.023)
PB × TR	−0.001	−0.42	0.669 (−0.002, 0.001)	0.000	0.02	0.984 (−0.001, 0.002)
PB × IN	−0.006	−2.61	0.009 (−0.010, 0.001)	0.004	2.02	0.043 (0.001, 0.008)
TR × IN	−0.047	−3.69	<0.001 (−0.072, −0.022)	−0.018	−1.56	0.116 (−0.041, 0.005)
PB × TR × IN	0.000	0.07	0.938 (−0.001, 0.001)	0.000	0.34	0.730 (−0.001, 0.001)
TR ± ME						
PB	0.035	7.36	<0.001 (0.026, 0.045)	0.032	7.52	<0.001 (0.023, 0.040)
TR	0.094	2.56	0.010 (0.022, 0.165)	0.282	8.68	<0.001 (0.218, 0.345)
ME	1.092	14.86	<0.001 (0.948, 1.236)	0.700	10.72	<0.001 (0.572, 0.828)
PB × TR	−0.002	−1.49	0.133 (−0.004, 0.001)	−0.000	−0.48	0.626 (−0.002, 0.001)
PB × ME	−0.001	−0.36	0.714 (−0.005, 0.004)	0.004	1.85	0.063 (−0.001, 0.008)
TR × ME	−0.008	−0.70	0.479 (−0.029, 0.013)	−0.032	−3.28	0.001 (−0.051, −0.013)
PB × TR × ME	−0.001	−1.30	0.190 (−0.001, 0.001)	0.010	2.14	0.032 (0.001, 0.010)
TR ± DE						
PB	0.028	6.55	<0.001 (0.019, 0.036)	0.028	7.29	<0.001 (0.021, 0.036)
TR	−0.079	−2.55	0.010 (−0.139, −0.018)	0.159	5.55	<0.001 (0.103, 0.215)
DE	1.806	30.95	<0.001 (1.692, 1.921)	1.186	21.77	<0.001 (1.079, 1.292)
PB × TR	−0.001	−1.15	0.249 (−0.003, 0.001)	−0.001	−1.44	0.149 (−0.002, 0.001)
PB × DE	−0.003	−1.83	0.066 (−0.007, 0.001)	0.005	3.00	0.002 (0.001, 0.009)
TR × DE	−0.030	−3.24	0.001 (−0.048, −0.012)	−0.031	−3.74	<0.001 (−0.048, −0.015)
PB × TR × DE	0.000	−0.10	0.918 (−0.000, 0.001)	0.010	2.36	0.017 (0.001, 0.010)

PB, Paranormal Belief; TR, Transliminality; UE, Unusual Experiences; CD, Cognitive Disorganisation; IA, Introvertive Anhedonia; IN, Impulsive Non-Conformity; ME, Manic Experience; DE, Depressive Experience.

**Figure 1:**
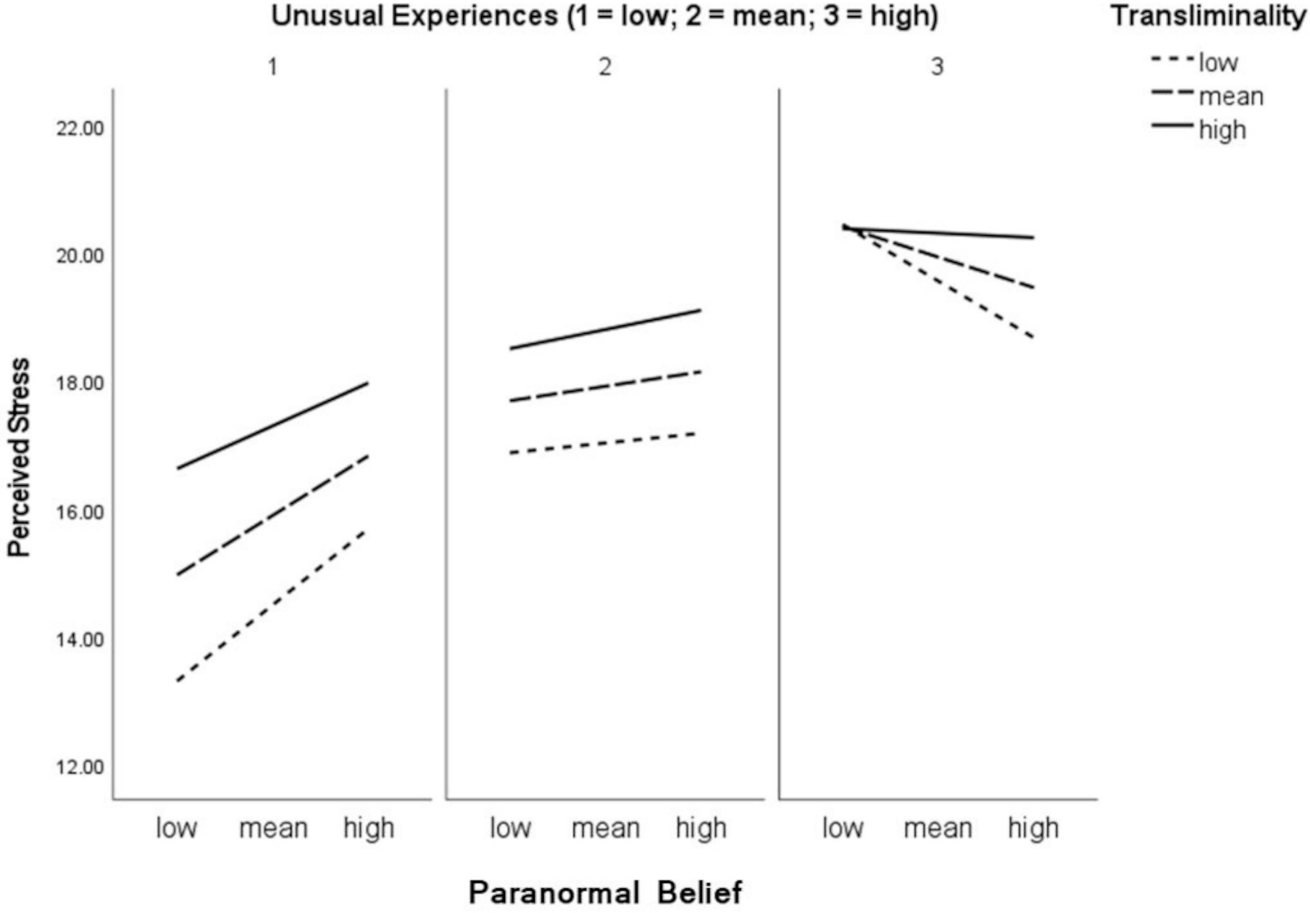
Paranormal Belief × Transliminality × Unusual Experiences predict Perceived Stress.

The inclusion of psychopathology-related variables to the moderation analyses revealed diverse effects (Table 2). For instance, with Unusual Experiences as a moderator, the direct and interactional relationship concerning Paranormal Belief, Transliminality and Perceived Stress became non-significant. In addition, a significant Paranormal Belief × Unusual Experiences, Transliminality × Unusual Experiences, and Paranormal Belief × Transliminality × Unusual Experiences interaction existed. Figure 1 displays the three-way interaction. Simple slopes indicated that, relative to this three-way interaction, the Paranormal Belief and Perceived Stress relationship was significant at low levels of Transliminality and Unusual Experiences (*t* = 5.09, *p* < 0.001), and at mean levels of Transliminality and low levels of Unusual Experiences (*t* = 3.68, *p* = 0.002). At mean and high levels of Unusual Experiences and high levels of Transliminality, however, a significant relationship did not exist. The model accounted for 14.2% of Perceived Stress variance.

**Figure 2:**
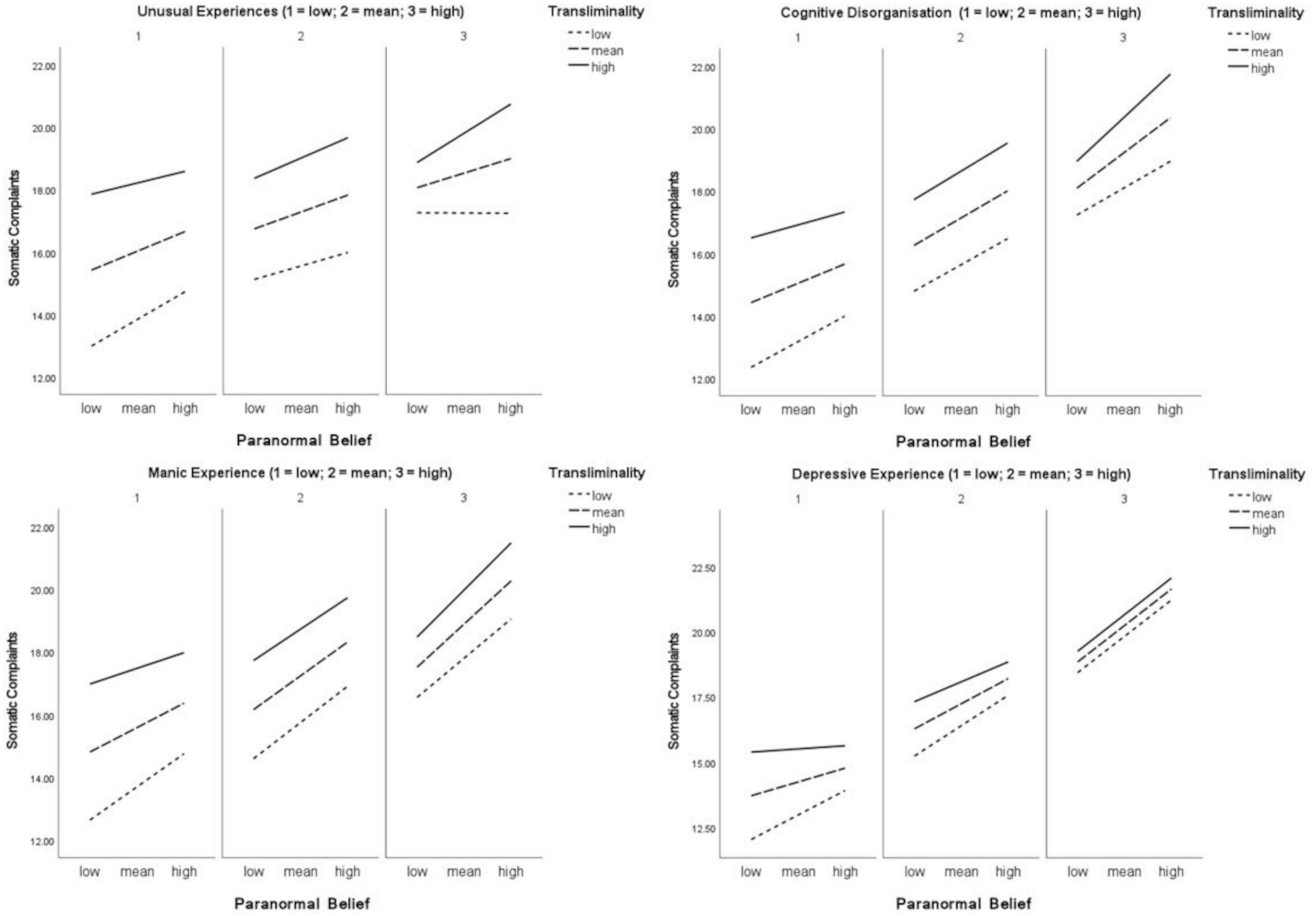
Paranormal Belief × Transliminality × psychopathology-related variables predict Somatic Complaints.

With Somatic Complaints as the criterion, Unusual Experiences was a significant predictor alongside Paranormal Belief and Transliminality. Moreover, a significant Transliminality × Unusual Experiences, and a three-way Paranormal Belief × Transliminality × Unusual Experiences interaction occurred (Figure 2). This indicated that among higher Transliminality and Unusual Experiences, higher Paranormal Belief aligned with increased Somatic Complaints. This trend was particularly noticeable at the higher level (>1SD) of Unusual Experiences. Explicitly, simple slopes suggested that the Paranormal Belief and Somatic Complaints relationship was significant at low levels of Transliminality and Unusual Experiences (*t* = 4.21, *p* < 0.001), at mean levels of Transliminality and low levels of Unusual Experiences (*t* = 2.76, *p* = 0.005), at mean levels of Transliminality and Unusual experiences (*t* = 3.43, *p* = <0.001), at high Transliminality and mean Unusual Experiences (*t* = 2.70, *p* = 0.006), and most prominently at high levels of both variables (*t* = 4.37, *p* < 0.001). In total, 22.2% of Somatic Complaints variance was accounted for.

Cognitive Disorganisation was a significant predictor of Perceived Stress, and meaningfully interacted with Paranormal Belief in terms of aligning with increased Perceived Stress. Though Transliminality and the three-way interaction was not significant, the overall model explained 31% of Perceived Stress variance. Similar findings occurred in relation to Somatic Complaints as for Unusual Experiences, in the sense Cognitive Disorganisation was a significant predictor and a significant Transliminality × Cognitive Disorganisation, and a three-way interaction existed (Figure 2). The interaction was prominent at the higher level of Cognitive Disorganisation (>1 SD). Specifically, simple slopes indicated that the Paranormal Belief and Somatic Complaints relationship was significant at low levels of Transliminality and low (*t* = 4.08, *p* < 0.001), medium (*t* = 4.62, *p* < 0.001), and high Cognitive Disorganisation (*t* = 2.89, *p* < 0.001). The Paranormal Belief-Somatic Complaints association was additionally significant at mean levels of Transliminality and low (*t* = 3.23, *p* = 0.001), medium (*t* = 6.42, *p* < 0.001), and high Cognitive Disorganisation (*t* = 5.61, *p* < 0.001). At high Transliminality and mean Cognitive Disorganisation (*t* = 4.59, *p* < 0.001) the association was significant, and it was most notable at high levels of both moderators (*t* = 6.94, *p* < 0.001). Overall, 27.5% of Somatic Complaints variance was explained.

Introvertive Anhedonia, although a significant predictor of Perceived Stress, did not significantly moderate its relationship with Paranormal Belief. Impulsive Non-Conformity exhibited a significant relationship with Perceived Stress and interacted with Transliminality and Paranormal Belief individually, but no three-way interaction occurred. Inclusion of Impulsive Non-Conformity resulted in a model accounting for 25.65% of Perceived Stress. Relative to Somatic Complaints, inclusion of Introvertive Anhedonia and Impulsive Non-Conformity produced similar results. Explicitly, both significantly predicted Somatic Complaints and interacted with Paranormal Belief, yet no meaningful three-way interaction ensued. The Introvertive Anhedonia model accounted for 24.86% of the variance, and the Impulsive Non-Conformity model explained 26.21%.

Manic Experience did not exert meaningful effects on the Paranormal Belief × Transliminality × Perceived Stress relationship (aside from predicting higher stress). However, for Somatic Complaints, Manic Experience was a significant predictor, and significant Transliminality × Manic Experience, and Paranormal Belief × Transliminality × Manic Experience interactions existed. The three-way interaction indicated that among higher Transliminality and Manic Experience, higher Paranormal Belief aligned with increased Somatic Complaints. Pertinently, simple slopes reported a significant Paranormal Belief and Somatic Complaints relationship at low Transliminality and low (*t* = 5.32, *p* < 0.001), medium (*t* = 5.88, *p* < 0.001), and high Manic Experience (*t* = 3.84, *p* < 0.001). The Paranormal Belief-Somatic Complaints association was furthermore significant at mean Transliminality and low (*t* = 3.60, *p* = 0.001), medium (*t* = 7.52, *p* < 0.001), and high Manic Experience (*t* = 6.38, *p* < 0.001). At high Transliminality and mean Manic Experience (*t* = 4.40, *p* < 0.001) the relationship was significant, and it was strongest at high levels of both moderators (*t* = 7.73, *p* < 0.001). The model accounted for 24.33% of Somatic Complaints variance.

For Depressive Experience, this significantly predicted Perceived Stress, and interacted with Transliminality. Yet a three-way interaction was not present. In total, the model accounted for 33.59% variance. In relation to Somatic Complaints, Depressive Experience was a significant predictor, and significant Paranormal Belief × Depressive Experience, Transliminality × Depressive Experience, and Paranormal Belief × Transliminality × Depressive Experience interactions existed. The three-way interaction (Figure 2) suggested that among higher Transliminality and Depressive Experience, Paranormal Belief associated with increased Somatic Complaints. Particularly, simple slopes exhibited a significant Paranormal Belief and Somatic Complaints association at low Transliminality and low (*t* = 4.84, *p* < 0.001), medium (*t* = 6.51, *p* < 0.001), and high Depressive Experience (*t* = 4.73, *p* < 0.001). The Paranormal Belief-Somatic Complaints relationship was also meaningful at mean Transliminality and low (*t* = 2.74, *p* = 0.006), medium (*t* = 7.29, *p* < 0.001), and high Depressive Experience (*t* = 7.03, *p* < 0.001). At high Transliminality and mean Depressive Experience (*t* = 3.73, *p* < 0.001) the association was significant, and it was most evident at high levels of both moderators (*t* = 7.59, *p* < 0.001). The model explained 32.68% of Somatic Complaints variance.

## Discussion

Paranormal Belief correlated positively with Transliminality, psychopathology-related measures (Schizotypy and Manic-Depressive Experience), and well-being (Perceived Stress and Somatic Complaints). Consideration of zero-order correlations using Gignac’s effect size guidelines for individual differences (Gignac and Szodorai, 2016) indicated that the associations with Transliminality and the Unusual Experiences subscale of the O-LIFE were large (0.30 and above), and the remaining relationships were in the medium range (0.20–0.29).

The observed relationships between Paranormal Belief and the measures were consistent with previous research (Transliminality, e.g., Houran et al., 2002; Unusual Experiences, e.g., Dagnall et al., 2016a; Manic-Depressive Experience, Thalbourne and French, 1995). Regarding positive associations between Paranormal Belief and higher levels of Perceived Stress and Somatic Complaints, these provided support for the supposition that supernatural credence within general populations is not an effective mechanism for resolving psychological and physical discomfort (Irwin, 2009; Roe and Bell, 2016). However, additional work is required because the present study considered only two narrow domains of well-being. This is especially necessary given the inconsistent outcomes previously observed within this research domain.

Moderation analysis found that as levels of Transliminality and Unusual Experiences increased, the strength of the Paranormal Belief and Perceived Stress relationship increased. Moreover, higher scores on Transliminality and psychopathology-related variables (i.e., Unusual Experiences, Cognitive Disorganisation, Manic Experiences, and Depressive Experiences) were associated with a stronger relationship between Paranormal Belief and Somatic Complaints.

Collectively, these outcomes indicated that Transliminality and psychopathology-related variables interacted with Paranormal Belief and were allied to lower levels of well-being. Explicitly, concurrent with heightened levels of Transliminality, Unusual Experiences predicted greater Perceived Stress. Similarly, alongside higher Transliminality, Unusual Experiences, Cognitive Disorganisation, Manic Experiences, and Depressive Experiences predicted higher levels of Somatic Complaints. These complex moderation effects may explain why preceding research, focusing on the direct influence of paranormal belief, has produced varied outcomes.

The observation that belief in the paranormal interacts with cognitive-perceptual personality factors in complex ways (Navarro and Guerra, 2020), which are differentially related to outcome measures such as well-being, concurs with recent studies using latent profile analysis (see Denovan et al., 2018; Drinkwater et al., 2022a). This emerging research designates that consideration of discrete, self-ascribed paranormal attributes (i.e., belief, experience, and ability) and cognitive-perceptual factors in isolation (i.e., schizotypy and transliminality) is too simplistic. This is particularly true of scales assessing paranormality (i.e., belief and experience) because they are restricted in focus and sample only limited ideation (e.g., RPBS – Revised Paranormal Belief Scale, Drinkwater et al., 2017).

Noting this, subsequent investigations should include more comprehensive measures that appraise a broader range of supernatural facets. In the case of belief, this could involve assessing wider construct content (Dagnall et al., 2010b), and/or combining factors such as belief, experience, and perceived ability. The advantage of this approach is that it recognizes that believers do not represent a homogenous group. Hence, rather than using the simple inter-group distinction between believers and non-believers, researchers could perform intra-group comparisons to determine whether certain profiles are more strongly related to well-being outcomes.

Commensurate with the notion that believers are best viewed as a heterogeneous group containing different sub-populations, within the present study Paranormal Belief in the absence of high scores on Transliminality and Schizotypy (especially Unusual Experiences) had a relatively benign influence on well-being. The importance of these constructs likely derives from the fact that they reference idiosyncratic variations in mentation (i.e., flow, structure, perception, and experience).

For instance, high Transliminality scores reflect a reduced ability to actively suppress irrelevant information from consciousness (Houran et al., 2006; Thalbourne, 2009b). This manifests as enhanced connectedness between mental processes and the spontaneous elicitation of emotions, cognitions, and behaviour (Lange et al., 2000). Regarding Unusual Experiences, the subscale encompasses items evaluating perceptual aberrations, magical thinking, and hallucinations (i.e., positive symptoms of psychosis). These are conceptualized as “additional” thoughts and feelings that extend “normal” experience (e.g., hallucinations and paranoia).

Considered in combination, these characteristics suggest that individuals high in Transliminality and Unusual Experiences are more sensitive to mental activity (due to lower sensory thresholds), place a greater emphasis on intrapsychic activity, and are less able to distinguish between internally and externally generated data (Dagnall et al., 2008). These cognitions are likely to promote awareness of psychological and physiological fluctuations. This interpretation concurs with several lines of investigation. Particularly, studies reporting strong associations between paranormal belief, schizotypy, deficits in reality testing, and a preference for intuitive thinking (Escolà-Gascón, 2020a,b). Collectively, this body of research in tandem with the current study, suggests that cognitive-perceptual factors interact with belief in complex ways and that these only become apparent when analysis considers the influence of indirect and moderating effects (Dagnall et al., 2015).

Although, the present study is correlational and causation cannot be inferred, within the framework of preceding work it is logical to infer that increased focus on and preoccupation with spontaneous cognitions and perceptions is likely to express as a perceived lack of control, and manifest as higher scores on negative well-being outcomes (i.e., perceived stress and somatic complaints). In the case of somatic complaints, the added contribution of Cognitive Disorganisation, and Manic-Depressive Experiences suggests that affect and disjointed cognitions play a role in increased levels.

This interpretation is consistent with previous studies that have noted relationships between depressive and anxiety disorders (Kroenke, 2003) and negative emotions (Terwogt et al., 2006). Moreover, research has shown that transliminality correlates with increased somatic complaints (in the form of somatic-hypochondriacal tendencies) in the context of paranormal belief (Houran et al., 2002). Thus, ensuing work should further explore the role that paranormal belief plays in the development of somatic complaints.

Although the findings of this investigation concurred with preceding academic work it is important to note some limitations. Firstly, the study used a cross-sectional design where data were collected at one point in time. This is problematic because single measurement points provide only brief snapshots of complex phenomena (i.e., beliefs, cognitive-perceptual features, and well-being). To assess the stability of effects over time, longitudinal follow-up studies with multiple comparison points are required. Explicitly, analysis of the lagged effects of paranormal belief and well-being could examine how the moderating effects of transliminality and schizotypy develop across time. This will determine the robustness and generality of effects. It will also suggest to investigators points at which interventions to counter negative well-being would be most effective.

Secondly, well-being was assessed only in terms of Perceived Stress and Somatic Complaints. Whilst, these are established, frequently employed measures they evaluate only particular aspects of psychological health. Noting this, ensuing investigations should employ a wider range of measures. These could include instruments such as the Psychological Wellbeing Scale (Ryff and Keyes, 1995), which measures autonomy, environmental mastery, personal growth, positive relations with others, purpose in life, and self-acceptance. Additionally, it would be worthwhile considering psychological distress. There is debate as to how this relates to well-being (Winefield et al., 2012). Although, well-being and ill-being share common factors, positive and negative affect are often viewed as independent (Winefield et al., 2012). Thus, future papers should consider whether well-being effects generalize to distress. This would produce a broader conceptualization of psychological adjustment. Lastly, given this study was exploratory, and a relatively large number of variables were assessed as potential moderators, it would be useful for subsequent research to replicate the findings with independent samples.

Further work is necessary since previous exploration of the relationship between belief in the paranormal and well-being has been somewhat simplistic and reductionist. This is true to the extent that, researchers have used limited measures of paranormality, considered only direct effects, and overlooked the relatively high incidence of paranormal endorsement within general populations. Considering the latter point, with hindsight it is not surprising that supernatural credence in isolation is not highly predictive of mental and physical health within non-clinical populations. Hence, only by considering broader aspects of paranormal ideation and how it interacts with cognitive-perceptual personal factors related to psychopathy, will theorists develop a sophisticated understanding of the conditions under which paranormal ascriptions are harmful or beneficial to well-being.

## Data availability statement

The raw data supporting the conclusions of this article will be made available by the authors, without undue reservation.

## Ethics statement

The studies involving human participants were reviewed and approved by the Manchester Metropolitan University Faculty of Health, Psychology and Social Care Ethics Committee granted ethical approval (December 2020; Project ID, 25390). The patients/participants provided their written informed consent to participate in this study.

## Author contributions

ND and AD provided theoretical focus, developed content, and produced the initial article. AD was responsible for data collection and measurement selection, performed data analysis, and wrote up the results. ND produced the discussion. ÁE-G contributed further analytical advice and support. ÁE-G and KD offered additional conceptual input. All authors contributed to the article and approved the submitted version.

## Funding

This research was supported by the BIAL Foundation (Project ID: 123/2020). The funder was not involved in the study design, collection, analysis, interpretation of data, the writing of this article, or the decision to submit it for publication.

## Conflict of interest

The authors declare that the research was conducted in the absence of any commercial or financial relationships that could be construed as a potential conflict of interest.

## Publisher’s note

All claims expressed in this article are solely those of the authors and do not necessarily represent those of their affiliated organizations, or those of the publisher, the editors and the reviewers. Any product that may be evaluated in this article, or claim that may be made by its manufacturer, is not guaranteed or endorsed by the publisher.
